# Performance Analysis of a Lower Limb Multi Joint Angle Sensor Using CYTOP Fiber: Influence of Light Source Wavelength and Angular Velocity Compensation

**DOI:** 10.3390/s20020326

**Published:** 2020-01-07

**Authors:** Letícia Avellar, Arnaldo Leal-Junior, Carlos Marques, Anselmo Frizera

**Affiliations:** 1 Graduate Program in Electrical Engineering, Federal University of Espirito Santo, 29075-910 Vitoria, Brazil; frizera@ieee.org; 2 Mechanical Engineering Department, Federal University of Espirito Santo, 29075-910 Espirito Santo, Brazil; leal-junior.arnaldo@ieee.org; 3 I3N & Physics Department, University of Aveiro, Campus Universitário de Santiago, 3810-193 Aveiro, Portugal; carlos.marques@ua.pt

**Keywords:** movement analysis, optical fiber sensors, joint angle, cytop

## Abstract

This paper presents the analysis of an intensity variation polymer optical fiber (POF)-based angle sensor performance, i.e., sensitivity, hysteresis and determination coefficient ($R^{2}$), using cyclic transparent optical polymer (CYTOP) fiber. The analysis consisted of two approaches: influence of different light source central wavelengths (430 nm, 530 nm, 660 nm, 870 nm and 950 nm) and influence of different angular velocities (0.70 rad/s, 0.87 rad/s, 1.16 rad/s, 1.75 rad/s and 3.49 rad/s). The first approach aimed to select the source which resulted in the most suitable performance regarding highest sensitivity and linearity while maintaining lowest hysteresis, through the figure of merit. Thereafter, the analysis of different angular velocities was performed to evaluate the influence of velocity in the curvature sensor performance. Then, a discrete angular velocity compensation was proposed in order to reduce the root-mean-square error (RMSE) of responses for different angular velocities. Ten tests for each analysis were performed with angular range of $0^{\circ }$ to $50^{\circ }$, based on knee and ankle angle range during the gait. The curvature sensor was applied in patterns simulating the knee and ankle during the gait. Results show repeatability and the best sensor performance for λ=950 nm in the first analysis and show high errors for high angular velocities (w=3.49 rad/s) in the second analysis, which presented up to 50% angular error. The uncompensated RMSE was high for all velocities ($6.45^{\circ }$ to $12.41^{\circ }$), whereas the compensated RMSE decreased up to 74% ($1.67^{\circ }$ to $3.62^{\circ }$). The compensated responses of application tests showed maximum error of $5.52^{\circ }$ and minimum of $1.06^{\circ }$, presenting a decrease of mean angular error up to $30^{\circ }$ when compared with uncompensated responses.

## 1. Introduction

The biomechanics of human movement are defined as the study of the human movement using methods of mechanical engineering [1]. The human movement analysis includes gait analysis, which comprises the systematic study of human walking, performed by collecting kinematic and kinect data, among them the joint angles [2,3]. In the clinical field, changes of the joint angles in the normal gait pattern may reveal key information about person’s quality of life [4]. The traditional scales used to analyze joint angles in clinical field are semi-subjective, based on specialists observation of the gait performance. It may provide a subjective evaluation of the quality of the patient’s gait with low accuracy and precision, which can result in diagnosis errors [4]. In contrast, the overwhelming advance in new technologies has facilitated the development of accurate and reliable devices and techniques which allow an objective evaluation of joint angles [5]. However, the technologies commonly used for joint angle measurement in gait analysis have some limitations. For example, the optical motion capturing systems which are expensive and require substantial dedicated space to be set up [6], restricting to indoor applications, and the inertial sensors which have lower accuracy and electromagnetic interference [7].

Optical fiber sensors (OFS) present advantages such as being lightweight, compact, chemically stabile, immune to electromagnetic fields, and for their multiplexing capabilities [8]. In addition, these sensors are attractive option for wearable applications, especially for joint angle measurement, due to their lower dimensions and flexibility and ability to be embedded in clothing or flexible structures [9,10].

Among the optical fiber materials, polymer optical fibers (POF) provide a much lower cost alternative to other optical fibers, besides the high elastic strain limits, high fracture toughness, high flexibility in bending and high sensitivity to strain. Polymers also have biocompatibility, favoring the use for biomedical applications [8]. For these reasons, POF sensors are an interesting alternative for the instrumentation of wearable devices for rehabilitation [7,11,12,13] and gait assistance [14].

To maintain the low cost, simpler interrogation techniques are usually used, such as the intensity variation principle. However, this technique offers precision limitations, presenting low sensitivity and systematic errors, such as nonlinearity, offset and hysteresis [15], when compared with more sophisticated measuring principles, such as nonlinear effects [16], fiber Bragg gratings (FBGs) [17] and interferometers [18].

The fabrication of POFs consists of a variety of optical polymers, including polymethyl-methacrylate (PMMA), cyclic transparent optical polymer (CYTOP produced by Chromis Fiberoptics, New Jersey, USA), cyclic olefin copolymer (COC), polystyrene (PS) and polycarbonate (PC) [8,19]. PMMA is the most common and popular material with a low cost [17]. However CYTOP has the lowest transmission loss among them [8,20], from the visible to near-infrared spectrum, which is a good option for sensing applications [9]. Moreover, CYTOP fibers present lower Young’s Modulus, which means higher flexibility [21] and lower hysteresis, when used as sensor, than PMMA [22].

Since the polymers are viscoelastic materials, they do not have constant response to stress and strain, especially in dynamic loading conditions [22]. The polymer response under a constant stress or strain dependents on the time that the load is applied (or removed), which can result in different responses under different angular velocities and accelerations [9], and can restrict the application in a small velocity range. To overcome these limitations and expand the application for any angular velocity, compensation techniques are proposed, such as [7] in which a hysteresis compensation was developed based on curves relating hysteresis and the angular velocity of the sensor. In [23], an error compensation was also applied in order to reduce the errors of the POF sensor caused by the hysteresis compensation. Exponential regression-based compensation models were also proposed to minimize the hysteresis caused by the variation on sensor linearity due to the polymer relaxation [24].

This paper presents the development, characterization and analysis of a lower limb multi joint angle POF-based sensor using CYTOP fiber and the proposal of an angular velocity compensation technique to reduce errors of the proposed sensor for gait application. Curvature tests were performed under different light source central wavelength and different angular velocities (within knee and ankle velocity range) in order to evaluate the sensor performance based on three parameters: sensitivity, hysteresis and determination coefficient ($R^{2}$) with exponential regression of each condition. This work proposes a sensor without lateral section due to the flexibility of CYTOP fiber, simplifying the fabrication of the sensor.

The proposed angular velocity compensation technique related the sensor angular velocities with hysteresis and RMSE, and a statistical analysis was made to determine if significant differences existed in previously defined parameters between velocities groups. Finally, the proposed compensation technique was applied on the POF angle sensor in two tests: simulating the knee and ankle angle patterns during the gait using the light source central wavelength which results in the best response.

The remainder of this paper is organized as follows. Section 2 presents the materials and methods used for the fabrication and the analysis of the sensor, including the experimental procedures, the statistical analysis and the proposal of angular velocity compensation. The results and discussion of each analysis, as well as the comparison of the responses with and without compensation and the application in movement analysis are presented in Section 3. Finally, the final considerations and future works are discussed in Section 4.

## 2. Materials and Methods

The POF was made of a commercial gradient index multimode CYTOP fiber (Chromis Fiberoptics Inc, USA) with a core diameter of 120 μm, a cladding thickness of 20 μm and a polycarbonate overcladding. The light sources were five light emitting diodes (LEDs) with different central wavelengths (λ): 430 nm, 530 nm, 660 nm, 870 nm and 950 nm (IF-E92A, IF-E93, IF-E97, IF-E91D and IF-E91A, respectively, Industrial Fiber Optics, USA). The selected LEDs correspond to different central wavelength of visible light region (blue – 430 nm, green – 530 nm and red – 660 nm) and different central wavelength of infrared region (870 nm and 950 nm), and are commonly commercially available. The optical power was converted into an electrical signal using a phototransistor IF-D92 (Industrial Fiber Optics, USA) and the acquisition was made through a microcontroller FRMD-KL25Z (NXP Semiconductors, Netherlands), at a sampling rate of 100 Hz. The data was filtered through the moving average filter with span of 5%, in order to eliminate the outliers observed in the measurements. The angle and the angular velocity were controlled using a servo motor.

The sensor operation principle is based on intensity variation, in which the attenuation of the transmitted optical signal power is related to the bending angle of the fiber, i.e., the transmission loss occur due to radiation losses caused by macrobending and is proportional to the variance of optical fiber’s bending angle.

### 2.1. Experimental Procedures

**Figure 1:**
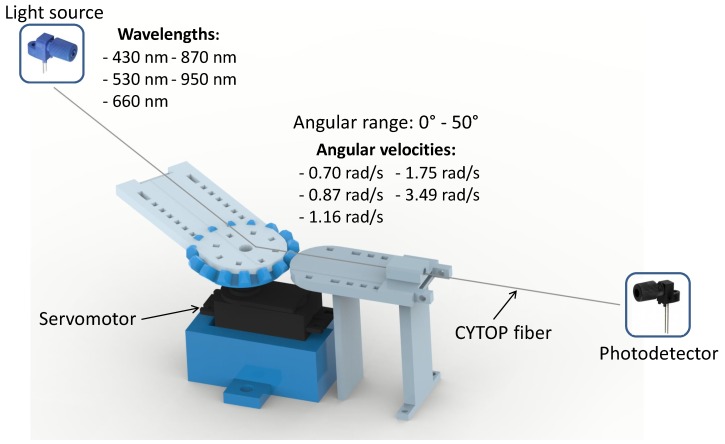
Experimental setup for evaluating the curvature sensor based on CYTOP fiber.

The experimental protocol was divided into two approaches in order to evaluate the performance of the CYTOP fiber under curvature: influence of different light source central wavelengths and influence of different angular velocities. Both approaches consisted of curvature tests with angular range from $0^{\circ }$ to $50^{\circ }$ and steps of $10^{\circ }$. This range was defined according to the knee and ankle angle patterns and limited at $50^{\circ }$ to ensure the reliability of the tests, due to servo motor limitation for high variation at high velocities. The analysis of the opposite bending ($-50^{\circ }$ to $0^{\circ }$) is not necessary, since the knee and ankle movements cannot be performed for the another direction due to the body limitation. However, it would be solved through lateral sections made in the fiber to create a sensitive zone. In the case of concave bending, there is an increase in reflections on the convex side of the curvature and a decrease on the concave side. Thus, if the lateral section is made in the concave side, the output power is higher for concave bending and lower for convex bending, and it is possible to identify the bending direction. Figure 1 shows the experimental setup for evaluation of the POF-based curvature sensing performance using CYTOP fiber.

In the first approach, curvature tests with angular velocity of 0.87 rad/s were performed. In addition, LEDs with different central wavelengths were employed to compare the responses related to wavelength. Sensitivity, hysteresis and $R^{2}$ were analyzed, and through these factors, the figure of merit (FoM) was calculated (as shown in Equation (1)) in order to characterize the wavelength which results in the higher FoM, consequently the best sensor performance. In Equation (1), *S* is the sensitivity, *h* is the hysteresis, $R^{2}$ is the determination coefficient, and α, β, θ are coefficients which define the weight of each performance factor.

(1) $$FoM=\alpha \cdot S-\beta \cdot h+\theta \cdot R^{2}$$

Thereafter, tests with different angular velocities (0.70 rad/s, 0.87 rad/s, 1.16 rad/s, 1.75 rad/s and 3.49 rad/s) and the light source central wavelength previously defined by FoM were conducted to evaluate the influence of velocity in the curvature sensor performance (sensitivity, hysteresis and $R^{2}$).

Based on these tests, an angular velocity compensation technique was proposed in order to reduce the root-mean-square error (RMSE) in different angular velocities (see Section 2.2). In both approaches, 10 tests were performed. Finally, the proposed compensation technique was applied on the POF angle sensor in two tests: simulating the knee and ankle patterns during the gait.

### 2.2. Statistical Analysis and Angular Velocity Compensation

A Shapiro–Wilk test was used to verify the data normality. Since the data are normal, one-way ANOVA (analysis of variance) was applied to determine if significant differences in sensitivity, hysteresis and angular error existed among different angular velocities with a significance level of 0.05, and the significant angular velocity groups were defined through this analysis.

**Figure 2:**
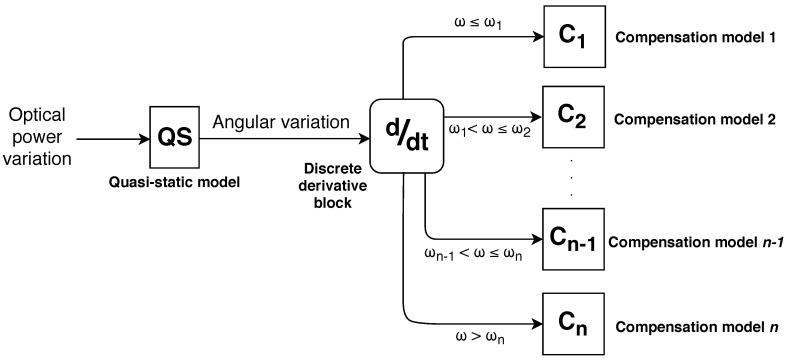
State machine diagram of the angular velocity compensation.

To reduce the RMSE of angle measurements, compensation models were developed for each angular velocity group. Figure 2 shows the state machine diagram describing the proposed angular velocity compensation, in which “QS” block comprises the model obtained on a quasi-static test, i.e., calibration curve with the lowest influence of the sensor angular velocity, and $C_{n}$ comprises the compensation models for respective angular velocity range.

Since the CYTOP is a viscoelastic material, its strain response depends on the time and the polymer relaxation can lead to the sensor hysteresis and possible variations on its linearity. To obtain a response close to the ideal (servo motor input), i.e., linear response, the compensation models were based on a sum of exponential with order 2, as shown in Equation (2), where $a_{n}$ are the model coefficients, α is the compensated angle (${}^{\circ }$) and *P* is the sensor power variation (ADC unit).

(2) $$\alpha =a_{1}\cdot e^{a_{2}\cdot P}+a_{3}\cdot e^{a_{4}\cdot P}$$

## 3. Results and Discussion

**Figure 3:**
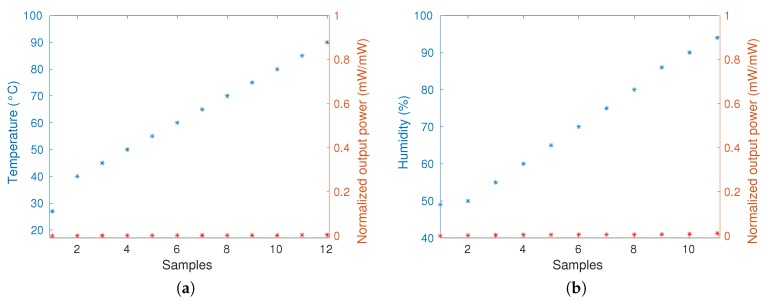
(**a**) Sensor response related to temperature variation. (**b**) Sensor response related to humidity variation.

To verify the stability of the sensor material related to temperature and humidity variation, two tests were performed. One test consisted of the sensor response analysis with the temperature increase and the other test consisted of the sensor response analysis with the increase of relative humidity. Figure 3a,b present the results of these tests, which showed that the temperature and the humidity do not significantly influence the sensor response. The pH was not evaluated in this work since this application is not influenced by this factor, because there is no pH variation in the environment where the sensor is positioned.

**Figure 4:**
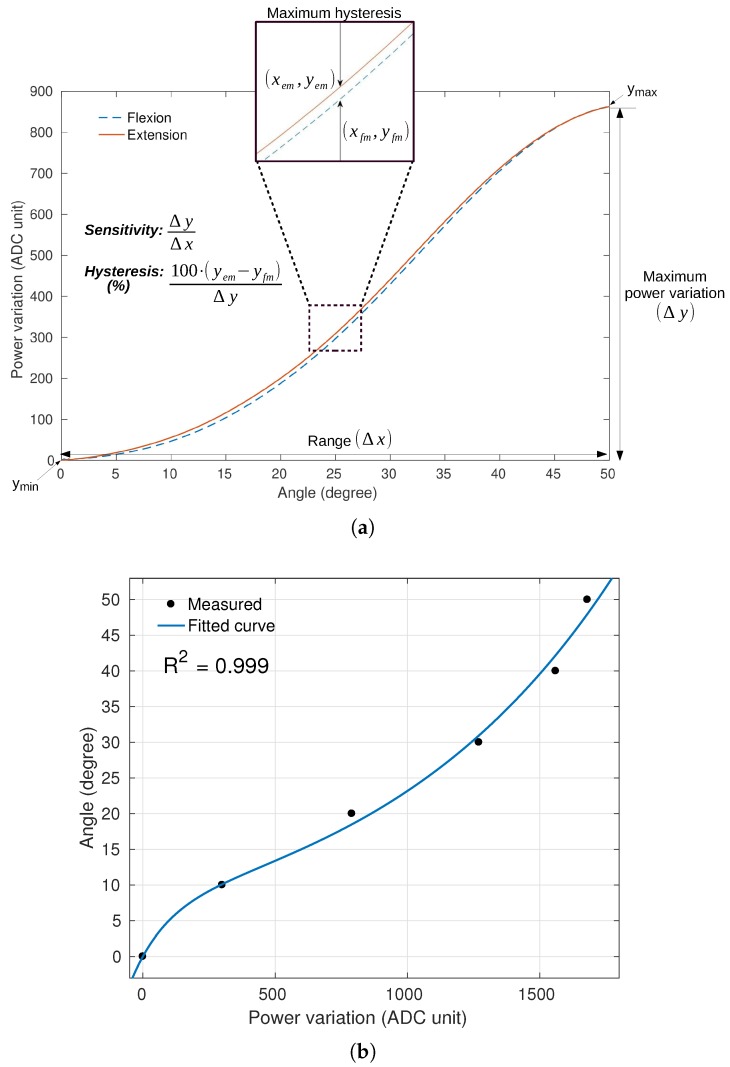
(**a**) Flexion and extension applied on CYTOP fiber showing the sensitivity and hysteresis concept. (**b**) Exponential regression of one curvature test showing the determination coefficient $R^{2}$.

The analysis of POF curvature sensor in CYTOP fiber is based on three performance factors: sensitivity, hysteresis and $R^{2}$. Figure 4a shows the concept of sensitivity and hysteresis applied in one result, where $R^{2}$ corresponds to the determination coefficient with an exponential regression, as shown in Figure 4b.

### 3.1. Analysis of Different Light Source Central Wavelengths

**Figure 5:**
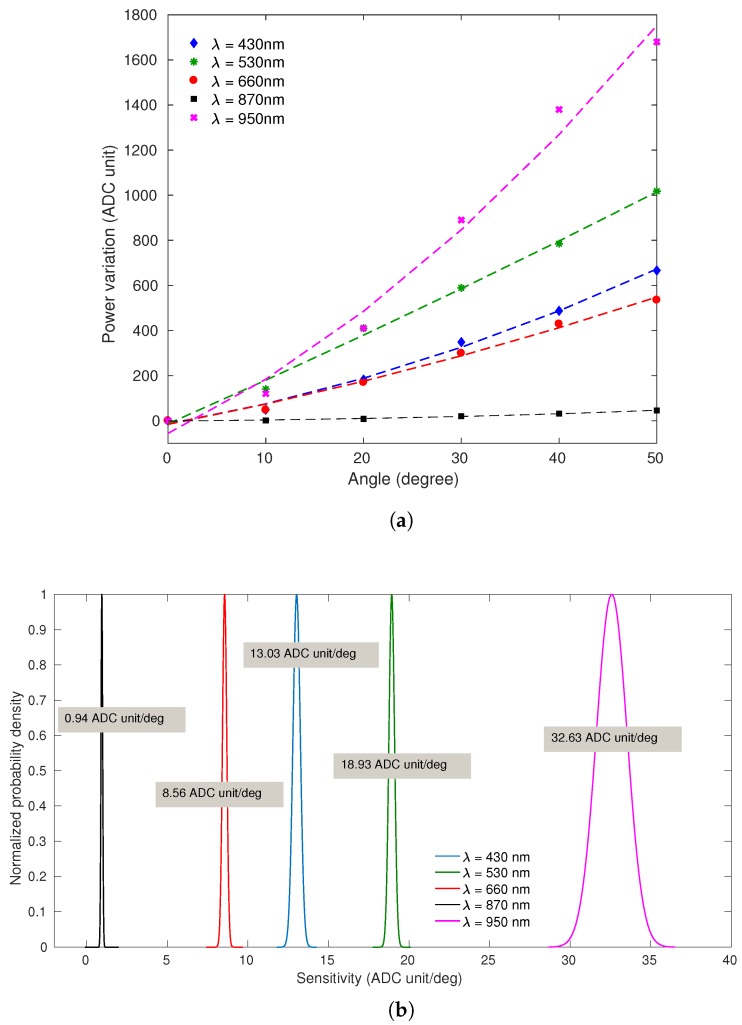
Sensor’s response in the curvature characterization with fitted curves: (**a**) Power variation of each central wavelength in angle range of 50 degrees. (**b**) Normal distribution of sensitivity for each central wavelength.

Figure 5a presents the sensors’ responses to the curvature applied to the CYTOP fiber (angular range from $0^{\circ }$ to $50^{\circ }$ and constant angular velocity of 0.87 rad/s) for different central wavelengths (λ), in which the markers represent the measured output and the dashed lines represent the sensors fit. The sensors’ responses presented exponential behavior with $R^{2}$ higher than 0.99 in all characterization tests. It is possible to observe the normal distribution of sensitivities in Figure 5b, in which the light source central wavelength of 950 nm provide the higher sensitivity (32.63 ADC unit/deg) of the sensor, whereas the light source central wavelength of 870 nm provide the lower sensitivity (0.94 ADC unit/deg). This may occur due to the some reasons, such as the decreasing optical attenuation curve of CYTOP fiber which presents higher attenuation for λ=430 nm and lower optical attenuation for λ=950 nm [8]. In addition, the photodetector as a function of the wavelength presents higher responsivity for 800 nm <λ<950 nm.

Thus, the sensor using LED with central wavelength of λ=950 nm presented highest sensitivity. The λ=870 nm should present the second highest sensitivity; however, the current of the tests is lower than the forward current presented in the light source datasheet [25]. Since the currents used in all tests has the same value and the λ=870 nm need a higher current value, the optical power is relatively lower, as the sensitivity when compared with others light source central wavelengths. In addition, the λ=660 nm presented the lower sensitivity among the visible spectrum (λ=430 nm, 530 nm, 660 nm) due to the lowest full-spectral bandwidth of the light source, resulting in a lower output power, since the photodetector acquires the integral of the light source spectrum, and, consequently, lower sensitivity.

Based on these performance factors, a FoM was applied to calculate the weighted sum of the factors and to select the central wavelength that results the higher FoM. The weighted coefficients for FoM calculation were defined as α=0.5, β=0.2, θ=0.3, since the sensitivity is a important factor which significantly decreases as the angular velocity increases, and hysteresis can be smoothed through some compensation technique [23]. Table 1 presents the results of each FoM, in which the light source central wavelength of 950 nm provides the higher FoM, being the best option for following applications.

### 3.2. Analysis of Different Angular Velocities

**Figure 6:**
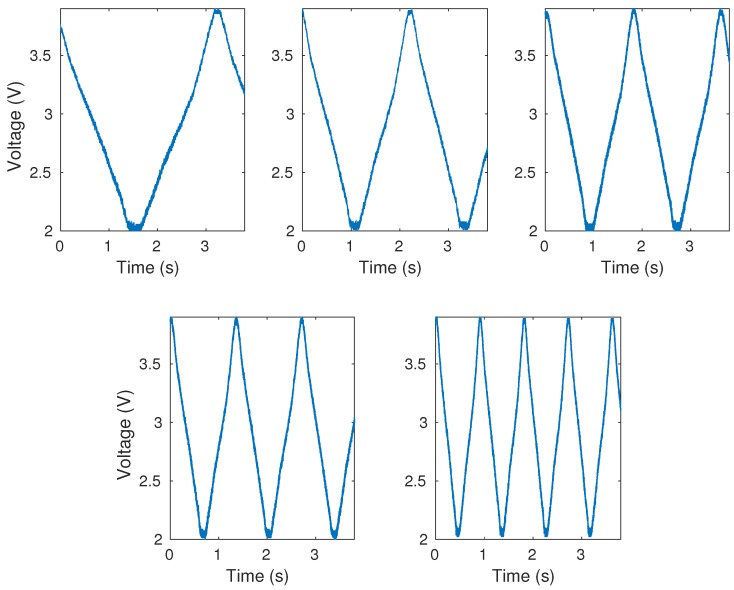
Raw measurements for each angular velocity.

After the first set of tests to select the light source central wavelength, tests with the angular range from $0^{\circ }$ to $50^{\circ }$ and different angular velocities are performed, as shown in Figure 1. Figure 6 shows the results of measurements without data treatment for the different angular velocities. All graphs show curvature cycles performed during the same time variation at different angular velocities. It is noticeable that the sensor responses present low noise; however, the moving average filter is used to eliminate the outliers observed in the measurements. The three performance factors (sensitivity, hysteresis and $R^{2}$) are analyzed, and Table 2 shows the mean and the standard deviation (SD) of sensitivity (ADC unit/deg), hysteresis (%) and angular error (${}^{\circ }$) of tests for each angular velocity.

**Figure 7:**
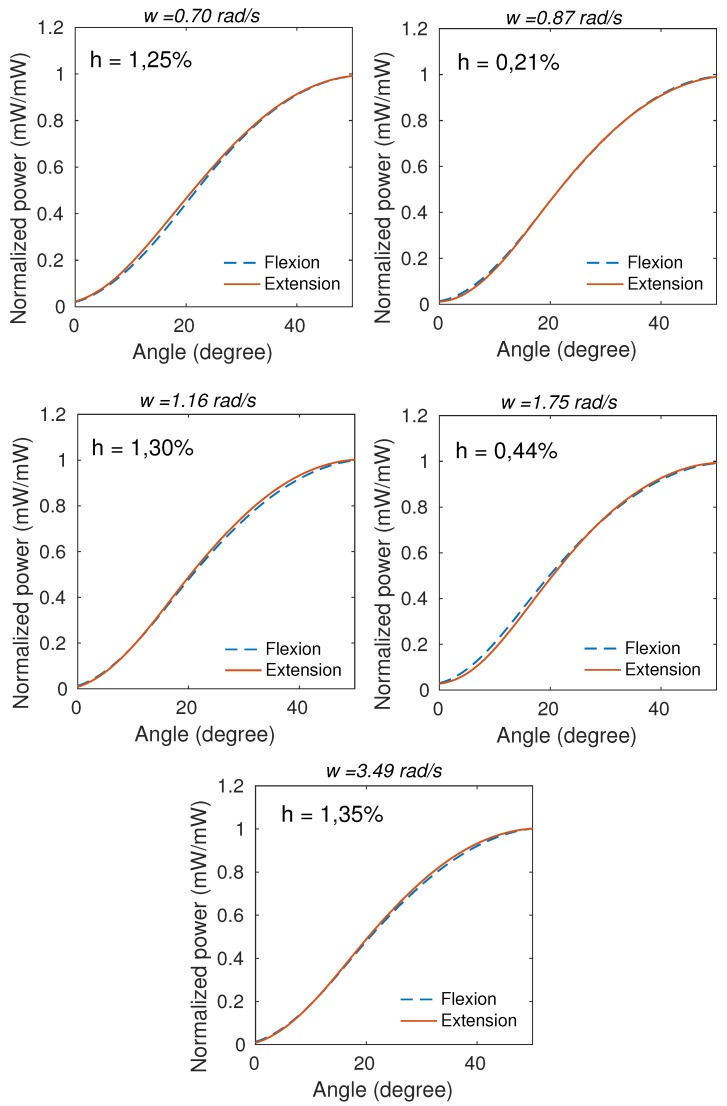
Mean of the cycles at each angle presenting the mean hysteresis for each angular velocity.

According to Table 2, it is noticeable that w=3.49 rad/s presented worst performance compared with others velocities, with lower sensitivity, equivalent to approximately 40% of the others, error of $25.25^{\circ }\left(2.00^{\circ }\right)$, corresponding to 50% of error and highest hysteresis of 3.12%(2.82%). Excluding this angular velocity (w=3.49 rad/s), all data are normal and the one-way ANOVA test showed that the sensitivities did not showed significant difference (p=0.1831), as well the hysteresis (p=0.1042) and the angular error (p=0.1841). In addition, the maximum angular error was of $1.31^{\circ }\left(0.77^{\circ }\right)$, which corresponds to 2.62% of error, and the minimum angular error was of $0.78^{\circ }\left(0.58^{\circ }\right)$, which corresponds to 1.56% of error. These results show that the sensor is repeatable and presents similar responses for this angular velocity range (0.70 to 1.75 rad/s) and as the velocity increases, the sensor performance decline. All angular velocities presented $R^{2}$ higher than 0.99. The mean of the cycles at each angle for all tested angular velocities presented low hysteresis, as shown in Figure 7, in which h is the hysteresis and ω is the angular velocity.

In addition to angular errors, the sensor responses presented high RMSE with maximum mean of $12.41^{\circ }\left(1.49^{\circ }\right)$ and minimum of $6.45^{\circ }\left(0.43^{\circ }\right)$. For this reason, the angular velocity compensation technique was applied in order to decrease the RMSE of angle measurements according to the angular velocity. Since the angular velocity range from 0.70 to 1.75 rad/s did not present significant differences, the angular velocity compensation technique was fitted for two groups: first group (angular velocities lower than or equal to 1.75 rad/s) and second group (angular velocities higher than 1.75 rad/s). The $C_{1}$ and $C_{2}$ blocks comprise the compensation models for two angular velocity groups. Equations (3)–(5) show the compensation models QS, C1 and C2 (see Figure 2) relating the optical power variation (P) with angle, respectively.

(3) $$QS\left(P\right)=16.43\cdot e^{-0.0007\cdot P}-16.40\cdot e^{-0.0061\cdot P}$$

(4) $$C_{1}\left(P\right)=8.93\cdot e^{0.0010\cdot P}-9.75\cdot e^{-0.0080\cdot P}$$

(5) $$C_{2}\left(P\right)=12.53\cdot e^{0.0025\cdot P}-12.84\cdot e^{-0.0091\cdot P}$$

**Figure 8:**
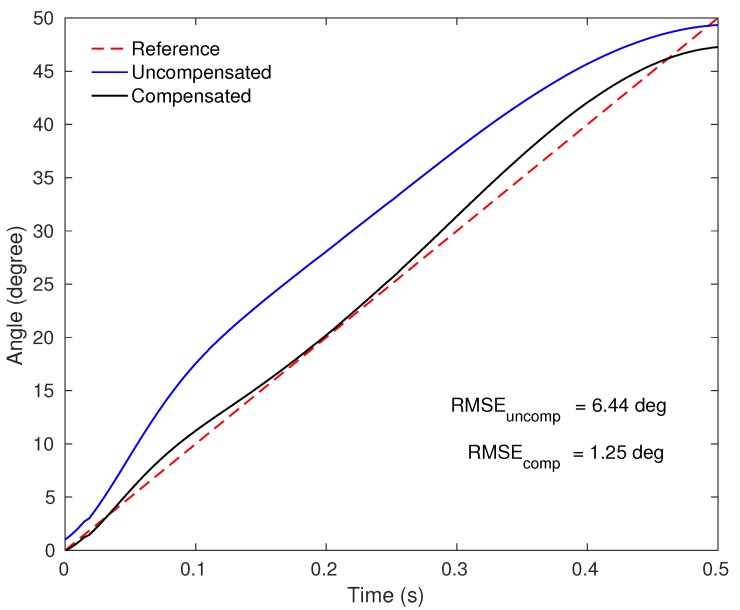
Angle curves with and without angular velocity compensation compared with the reference of one test.

Figure 8 shows the angle curves of characterized sensors responses in a quasi-static tests (uncompensated) and with the angular velocity compensation (compensated) compared to the servo motor input (reference) in one test, and Table 3 shows the RMSE of angle measurements for uncompensated and compensated response in each angular velocity.

The compensated responses presented a RMSE decrease up to 74% (w=1.16 rad/s), with minimum error of $1.67^{\circ }\left(0.27^{\circ }\right)$ for angular velocity of w=1.16 rad/s. The higher angular velocity (w=3.49 rad/s) presented two cycles with major differences from the others, resulting in RMSE $6.85^{\circ }\left(5.23^{\circ }\right)$. However, excluding these two cycles the RMSE was $3.62^{\circ }\left(1.32^{\circ }\right)$. Although the responses demonstrate worst performance in this angular velocity, the two different cycles may be related to servo motor performance at high velocities, presenting errors up to three times higher than the mean, which can be considered outliers.

### 3.3. Applications in Movement Analysis

**Figure 9:**
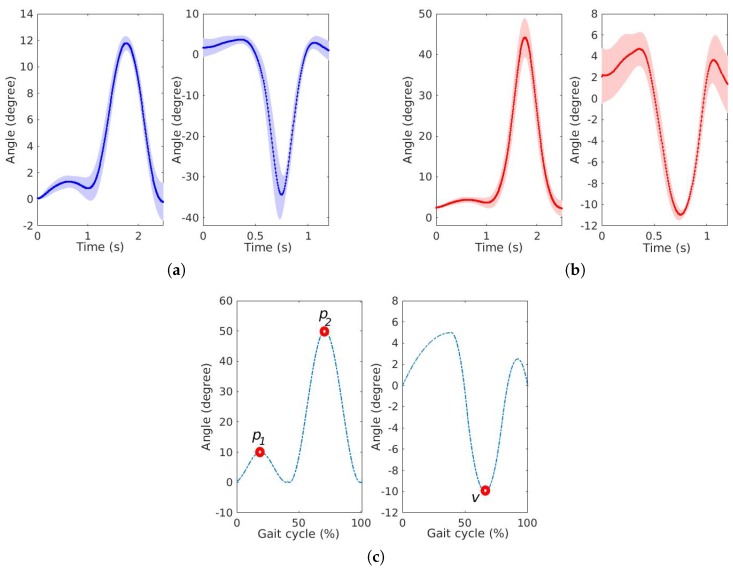
Results of sensor responses of knee and ankle simulating curves: (**a**) Uncompensated response. (**b**) Compensated response. (**c**) Reference patterns according to [26].

Figure 9 presents the results of uncompensated and compensated knee and ankle curves, where the continuous lines represent the mean of the cycles and the shaded lines represent the standard deviation of the cycles. The knee pattern curve presents two peaks ($p_{1}=10^{\circ }$ and $p_{2}=50^{\circ }$) and the ankle pattern curve presents one valley ($v=-10^{\circ }$). The uncompensated responses presented $p_{1}$ = $1.40^{\circ }\left(0.50^{\circ }\right)$, $p_{2}$ = $11.85^{\circ }\left(0.60^{\circ }\right)$ and *v* = $-35.54^{\circ }\left(6.64^{\circ }\right)$, resulting in errors of $8.60^{\circ }$, $38.15^{\circ }$ and $25.54^{\circ }$, respectively. The compensated responses presented $p_{1}$ = $4.48^{\circ }\left(0.78^{\circ }\right)$, $p_{2}$ = $44.80^{\circ }\left(5.44^{\circ }\right)$ and *v* = $-11.06^{\circ }\left(0.60^{\circ }\right)$, resulting in errors of $5.52^{\circ }$, $5.20^{\circ }$ and $1.06^{\circ }$, respectively. Table 4 shows the results of uncompensated and compensated responses for the two gait applications.

The high errors of uncompensated responses are due to the variation of angular velocity, resulting in a different response from the characterization. When the angular velocity compensation was applied, according to the angular velocity of the curves, the response was more similar to the reference, with a decrease of $30^{\circ }$ angular error for $p_{2}$, approximately.

## 4. Conclusions

This paper presented the performance analysis of a POF curvature sensor using CYTOP fiber under two conditions: different light source central wavelengths and different angular velocities. In addition, the sensor was applied on two approaches in the gait analysis: simulating knee and ankle patterns during the gait. The curvature sensor comprises of POF based on intensity variation principle, which present the lowest cost and highest simplicity in the fabrication and the signal processing. Results showed differences in sensitivity and hysteresis between light source central wavelengths, where best performance of λ=950 nm was obtained through FoM evaluation. In addition, results showed high RMSE for different velocities, especially under w=3.49 rad/s. The angular velocity compensation decreased the RMSE up to 74% and presented an improvement of responses in the gait simulating application, decreasing the error up to 86.37%$\left(32.95^{\circ }\right)$. Future works include the application of the sensor in a multiplexing technique to assess all joints simultaneously.

## Figures and Tables

**Table 1:** FoM of sensor performance for each central wavelength.

λ	Sensitivity	Hysteresis (%)	$R^{2}$	FoM
430 nm	13.03	1.78	0.997	6.81
530 nm	18.93	1.66	0.998	9.76
660 nm	8.56	1.44	0.998	4.58
870 nm	0.94	2.60	0.996	0.77
950 nm	32.63	2.73	0.993	16.58

**Table 2:** Mean(SD) of sensor performance factors for each angular velocity.

Angular Velocity	Sensitivity	Hysteresis (%)	Angular Error (${}^{\circ }$)
3.49 rad/s	12.85(2.14)	3.12(2.82)	25.25(2.00)
1.75 rad/s	33.01(0.48)	2.37(2.14)	1.31(0.77)
1.16 rad/s	33.33(0.38)	1.17(0.89)	0.82(0.55)
0.87 rad/s	32.63(0.39)	2.73(2.48)	1.16(0.63)
0.70 rad/s	33.33(0.36)	1.72(1.13)	0.78(0.58)

**Table 3:** Mean(SD) RMSE of angle measurements for each angular velocity with uncompensated and compensated responses.

Angular Velocity	Uncompensated	Compensated
3.49 rad/s	$12.41^{\circ }\left(1.49^{\circ }\right)$	$3.62^{\circ }\left(1.32^{\circ }\right)$ ${}^{1}$
1.75 rad/s	$6.93^{\circ }\left(1.30^{\circ }\right)$	$1.94^{\circ }\left(0.97^{\circ }\right)$
1.16 rad/s	$6.45^{\circ }\left(0.43^{\circ }\right)$	$1.67^{\circ }\left(0.27^{\circ }\right)$
0.87 rad/s	$6.90^{\circ }\left(1.07^{\circ }\right)$	$2.03^{\circ }\left(0.63^{\circ }\right)$
0.70 rad/s	$7.97^{\circ }\left(1.27^{\circ }\right)$	$2.33^{\circ }\left(0.72^{\circ }\right)$

${}^{1}$ Excluding outliers.

**Table 4:** Results of uncompensated and compensated responses for the two gait applications.

	Reference	Uncompensated		Compensated	
	Angle	Angle	Error	Angle	Error
$p_{1}$	$10^{\circ }$	$1.40^{\circ }\left(0.50^{\circ }\right)$	$8.60^{\circ }$	$4.48^{\circ }\left(0.78^{\circ }\right)$	$5.52^{\circ }$
$p_{2}$	$50^{\circ }$	$11.85^{\circ }\left(0.60^{\circ }\right)$	$38.15^{\circ }$	$44.80^{\circ }\left(5.44^{\circ }\right)$	$5.20^{\circ }$
*v*	$-10^{\circ }$	$-35.54^{\circ }\left(6.64^{\circ }\right)$	$25.54^{\circ }$	$-11.06^{\circ }\left(0.60^{\circ }\right)$	$1.06^{\circ }$
